# Therapeutic Potential of Specific *Lacticaseibacillus rhamnosus* Strains for DNCB-Induced Atopic Dermatitis in Mice

**DOI:** 10.3390/nu18091335

**Published:** 2026-04-23

**Authors:** Tingchao He, Qidong Lu, Jian Zhang, Xinyu Xie, Xin Liu, Hua Jiang, Jing Li, Yumei Zhang

**Affiliations:** 1 Department of Nutrition and Food Hygiene, School of Public Health, Peking University, Beijing 100191, China; 2 Department of Hygiene, Fujita Health University, Toyoake-shi 470-1192, Japan; 3 Mom’s Garden Institute of Nutrition and Health, Bismarckstraße 37, 66121 Saarbrücken, Germany; 4 School of Nursing, Peking University, Beijing 100191, China

**Keywords:** *Lacticaseibacillus rhamnosus*, atopic dermatitis, gut microbiota

## Abstract

**Background**: Atopic dermatitis (AD) is a chronic inflammatory skin disease linked to epidermal barrier dysfunction, Th2-skewed immune polarization, and disrupted gut microbiota homeostasis. While probiotic interventions show promise in managing AD, the mechanisms governing strain-specific efficacy—particularly systemic modulation via the “gut–skin axis”—remaining to be fully elucidated. **Methods**: This study systematically compared the oral therapeutic effects of three *Lacticaseibacillus rhamnosus* strains (MG-A047, MG-A054, and LGG) in a 2,4-dinitrochlorobenzene (DNCB)-induced AD mouse model. **Results**: By integrating behavioral, histopathological, and serological assessments with 16S rRNA-based gut microbiota profiling and in vitro functional assays, this study offers a multidimensional evaluation of the strain-specific advantages and potential therapeutic mechanisms of three *L. rhamnosus* strains. The results demonstrate that MG-A054 most effectively alleviated cutaneous inflammation and pruritus, significantly reduced serum IgE and IL-4 levels, and attenuated epidermal hyperplasia and inflammatory cell infiltration (including mast cells and eosinophils). Mechanistically, this strain may directly inhibit hyaluronidase activity and mast cell degranulation, and specifically remodel the gut microbiota structure, thereby promoting a shift toward a healthier functional profile. **Conclusions**: These findings suggest that the superior efficacy of MG-A054 may be achieved through coordinated modulation of the gut–skin axis and related pathways. This study offers new mechanistic clues for understanding the strain-specific actions of probiotics and lays a preclinical foundation for the further development of MG-A054 as a potential targeted microecological therapy for AD.

## 1. Introduction

AD is a chronic, relapsing inflammatory dermatosis characterized by hallmark symptoms such as xerosis, erythema, papules, intractable pruritus, and lichenification [1]. Epidemiological studies report a rising global prevalence, affecting approximately 10–20% of children and 1–3% of adults, with a higher incidence observed in females [2]. The pathogenesis of AD involves a complex interplay of genetic predisposition, immune dysregulation, skin barrier dysfunction, environmental exposures, and intestinal dysbiosis [3,4]. Emerging evidence implicates gut microbial imbalance—specifically, a reduction in beneficial bacteria and an overgrowth of opportunistic pathogens—as a key contributor to AD development and exacerbation [5]. Analyses of fecal microbiota from AD patients show a significant increase in the abundance of pathogenic bacteria within the Firmicutes phylum, such as *Staphylococcus aureus* [6], concurrent with a marked decrease in beneficial genera like *Bifidobacterium* and *Lactobacillus* [7]. Mechanistic studies further indicate that intestinal *Bifidobacterium* can mitigate skin inflammation by promoting short-chain fatty acid (e.g., acetate and propionate) production, suppressing Th2-type immune responses, and modulating the gut microbial community [8]. Based on these mechanisms, probiotic intervention has emerged as a promising strategy for managing AD. The primary mechanisms of action include modulating gut microbiota composition, enhancing intestinal epithelial barrier function (through upregulating tight-junction proteins and producing short-chain fatty acids), and regulating immune responses (such as suppressing pro-inflammatory cytokines like IL-4 and IL-17, promoting anti-inflammatory factors like IL-10 and TGF-β, expanding regulatory T cells, and inhibiting Th2 cell differentiation) [9]. Probiotics also suppress pathogen invasion and mast cell-mediated inflammation, thereby restoring immune homeostasis [10].

*L. rhamnosus*, a Gram-positive, facultative anaerobic lactic acid bacterium, commonly colonizes the gastrointestinal mucosa and is naturally found in fermented dairy products and cereal-based foods [11]. Due to its notable immunomodulatory, barrier-restorative, and anti-inflammatory properties, this species is considered a potential probiotic candidate for AD intervention, among which the strain *L. rhamnosus* GG (LGG) has been extensively studied [12]. LGG’s immunomodulatory effects are context-specific. In infants with AD, it alleviates symptoms by rebalancing gut microbiota and Th1/Th2 responses [13]. Conversely, in a birch pollen allergy model, it strengthens oral immunity by increasing salivary IgA [14]. In a cow’s milk allergy (CMA) model, LGG supplementation shifts the immune response from a Th2-dominant to a Th1-oriented profile [15]. These findings not only confirm the beneficial role of LGG in AD intervention but also suggest that *L. rhamnosus* may possess strain-specific immunomodulatory properties. In recent years, beyond LGG, other strains of *L. rhamnosus*, such as HN001 and IDCC 3201, have demonstrated potential in alleviating AD across different study populations. For instance, studies on *L. rhamnosus* HN001 indicate that this strain can systemically modulate immune responses by modulating gut microbiota composition, enhancing regulatory T cell (Treg) function, and balancing key cytokines such as interferon-γ (IFN-γ) and transforming growth factor-β (TGF-β), thereby conferring significant and sustained protection against AD in high-risk infants [16,17]. Notably, heat-inactivated *L. rhamnosus* IDCC 3201 has demonstrated clinical value in pediatric AD management. By reducing levels of eosinophil cationic protein (ECP), peripheral blood eosinophil counts, and interleukin-31 (IL-31), it significantly improved symptoms in children aged 1–12 years with moderate AD [18]. These findings collectively indicate that various *L. rhamnosus* strains, both viable and inactivated, can offer novel microbiome-based strategies for AD management through distinct immunomodulatory and anti-inflammatory pathways.

Current evidence indicates that probiotics can ameliorate AD; however, most related studies have focused on clinical efficacy observations or mechanistic investigations of individual strains. However, systematic comparative research linking the functional phenotypes of probiotics to their underlying molecular mechanisms remains scarce, particularly for strains of human origin. Therefore, this study selected two *L. rhamnosus* strains isolated from healthy children (MG-A047 and MG-A054), with the widely used LGG strain serving as a reference. To systematically compare the therapeutic effects of these strains and explore their mechanisms of action, an AD mouse model was established using 2,4-dinitrochlorobenzene (DNCB) induction, and a multidimensional evaluation framework integrating “probiotic intervention–immune modulation–gut microbiota profiling” was developed. Our research aims to achieve three main objectives: first, to elucidate strain-specific modulatory effects on AD and their underlying mechanisms; second, to build a theoretical basis for the precision use of probiotics in AD management; and third, to pioneer new strategies for microbiome-based treatment of immune-mediated skin diseases.

## 2. Materials and Methods

### 2.1. Bacterial Strains and Culture Conditions

*L. rhamnosus* MG-A047 was isolated from fecal samples of healthy children, whereas *L. rhamnosus* MG-A054 was isolated from saliva samples of healthy children. Species identification for both strains was confirmed by amplifying and sequencing the 16S rRNA gene, followed by sequence similarity analysis using BLAST (version 2.16.0) against the NCBI database. *L. rhamnosus* MG-A054 has been deposited in the China General Microbiological Culture Collection Center (CGMCC) under the accession number CGMCC 37221. The reference strain *L. rhamnosus* GG (LGG) was obtained from Chr. Hansen Holding A/S (Hørsholm, Denmark). All strains were processed into bacterial powders via fermentation. For oral gavage in mice, the powders were resuspended in normal saline to a final concentration of 1 × 10^9^ CFU/mL.

### 2.2. Construction of AD Model and Experimental Design

The animal experimental protocol was approved by the Committee of Peking University Health Science Center (Application No. DLASBE0689, approval date: 10 September 2025). Specific-pathogen-free (SPF) female Balb/c mice (8 weeks old, weighing 18–22 g) were purchased from Jiangsu Qinglongshan Biotechnology Co., Ltd. (laboratory animal license number: SCXK (Su) 2024-0001; Zhenjiang, China) and were housed under controlled conditions, including a 12 h light/dark cycle, a temperature of 25 ± 1 °C, and a relative humidity of 55 ± 10%.

**Figure 1:**
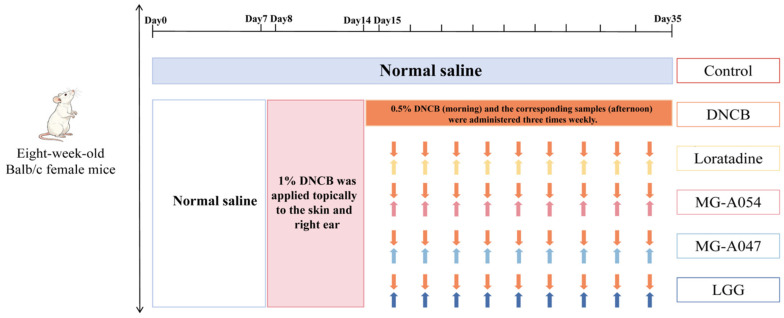
Experimental flowchart of animal treatments. Orange arrows: DNCB challenge (three times per week, in the morning). Colored arrows: Oral gavage of corresponding samples (three times per week, in the afternoon).

After one week of acclimatization, the mice were ranked by body weight and assigned to six groups (*n* = 12 per group) using a stratified randomization method based on previous studies. The sample size was determined based on sample sizes commonly used in comparable murine models of atopic dermatitis [19,20,21], which have consistently demonstrated sufficient statistical power to detect significant differences in key outcome measures such as serum IgE levels and histopathological scores. (1) Control: received daily oral gavage of 0.2 mL normal saline without DNCB sensitization; (2) DNCB: received daily oral gavage of 0.2 mL normal saline and was subjected to DNCB (Sinopharm Chemical Reagent Co. Ltd., Shanghai, China) sensitization; (3) Loratadine: Mice received loratadine (Xisimin, Peking University Pharmaceutical Co., Ltd., Beijing, China) via oral gavage at a dose of 15.17 mg/kg body weight on challenge days (three times per week). (4) LGG: administered *L. rhamnosus* GG suspension (1 × 10^9^ CFU/day in 0.2 mL) via gavage three times weekly for three weeks; (5) MG-A047: administered *L. rhamnosus* MG-A047 suspension (1 × 10^9^ CFU/day in 0.2 mL) via gavage three times weekly for three weeks; (6) MG-A054: administered *L. rhamnosus* MG-A054 suspension (1 × 10^9^ CFU/day in 0.2 mL) via gavage three times weekly for three weeks (Figure 1). All gavage solutions were prepared fresh in physiological saline daily. The sensitizing agent DNCB was dissolved in an acetone–olive oil mixture (3:1, *v*/*v*) to prepare 1% (*w*/*v*) and 0.5% (*w*/*v*) solutions. As outlined in Figure 1, the AD model was induced as follows: During the sensitization phase (days 8–11), 200 µL of 1% DNCB was applied to the shaved dorsal skin and 20 µL to the right ear pinna. The challenge phase (days 15–35) consisted of topical application of 200 µL (dorsal skin) and 20 µL (right ear) of 0.5% DNCB, administered three times per week. On challenge days, DNCB application was performed in the morning, followed by oral gavage administration of bacterial suspensions or loratadine in the afternoon. Throughout the experimental period, daily morning observations were conducted to assess changes in general health, activity levels, and signs indicative of pain or distress (e.g., spontaneous wiping behavior, abnormal posture, or vocalization). Body weight was recorded twice weekly. Humane endpoints were set as any of the following: body weight loss > 15%, severe skin ulceration or bleeding, reduced spontaneous locomotion, difficulty in eating or drinking, and overt signs of distress. Mice reaching any of these endpoints were immediately euthanized in accordance with animal welfare guidelines. During the experiment, all procedures, including model induction, drug administration, behavioral assessment, and histopathological scoring, were performed by investigators blinded to group allocation.

### 2.3. Symptom Assessment and Analysis

Following DNCB application, the severity of dorsal skin lesions was evaluated weekly in mice. The AD severity was scored based on four clinical signs: (1) erythema, (2) edema/papulation, (3) excoriation, and (4) lichenification. Each sign was graded on a scale of 0 (none), 1 (mild), 2 (moderate), and 3 (severe). To evaluate dermatitis severity, a total score (maximum 12) was derived from the sum of all individual component scores [22,23,24,25]. Scratching behavior was monitored weekly. For this purpose, by random selection, six mice per group were used for observation, and scratching bouts were recorded during a 30-min period starting one hour post-administration. Additionally, ear thickness was measured weekly at a consistent midpoint location using a digital thickness gauge. Assessment method: Each mouse was placed individually in a cage and allowed to acclimate for 10 min under quiet conditions (a smartphone may be used for video recording). Scratching episodes were counted. A scratching episode was defined as the mouse lifting its hind paw to scratch the lesional skin area continuously; a new episode was recorded when the mouse either lowered its paw or paused, followed by licking and subsequent scratching. Continuous scratching without interruption was counted as a single episode.

### 2.4. Histopathological Analysis

After blood collection, the newly regrown hair on the dorsal skin was shaved, and lesional skin tissue samples were collected. Tissues were fixed in 4% paraformaldehyde (Nanjing Friss Biotechnology Co., Ltd., Nanjing, China) according to standard protocols. Histopathological analysis was performed using hematoxylin and eosin (H&E) staining (Nanjing Friss Biotechnology Co., Ltd., Nanjing, China) to evaluate general pathological changes. Toluidine blue (Nanjing Friss Biotechnology Co., Ltd., Nanjing, China) and Congo red staining (Beijing Solarbio Science & Technology Co., Ltd., Beijing, China) were subsequently used to specifically assess mast cell and eosinophil infiltration, respectively. Histological analysis was performed using six mice per group (*n* = 6). For each mouse, multiple tissue sections were analyzed and averaged to obtain one value per animal for statistical analysis.

### 2.5. Biochemical Analysis

At the experimental endpoint, mice were euthanized, and whole blood was collected via retro-orbital puncture. The blood samples were allowed to clot at room temperature and then centrifuged at 3500× *g* for 15 min. The supernatant serum was carefully collected for subsequent analysis. Serum IgE levels were quantified using a commercial ELISA kit (Invitrogen, Carlsbad, CA, USA) strictly according to the manufacturer’s instructions. Serum IL-4 concentrations were measured using an IL-4-specific cytometric bead array (CBA).

### 2.6. Microbiota 16S rRNA Gene Sequencing

Following sample collection, total microbial DNA was extracted using the E.Z.N.A™ Mag-Bind Soil DNA Kit (Omega Bio-tek, Inc., Norcross, GA, USA) and quantified with a Qubit 4.0 fluorometer (Thermo Fisher Scientific, Waltham, MA, USA). The V3-V4 hypervariable region was amplified by PCR using universal primers and the 2× Hieff Robust PCR Master Mix (Yeasen Biotechnology (Shanghai) Co., Ltd., Shanghai, China), with the following cycling conditions: initial denaturation at 95 °C for 3 min; 5 cycles of denaturation at 95 °C for 30 s, annealing at 45 °C for 30 s, and extension at 72 °C for 30 s; 20 cycles of denaturation at 95 °C for 30 s, annealing at 55 °C for 30 s, and extension at 72 °C for 30 s; followed by a final extension at 72 °C for 5 min. The PCR products were purified by 2% agarose gel electrophoresis, followed by library construction (Shanghai, China) and sequencing on the Illumina MiSeq platform (Illumina MiSeq, San Diego, CA, USA). The sequencing data were processed by merging paired-end reads with PEAR (version 0.9.8) and clustering into operational taxonomic units (OTUs) at 97% similarity using USEARCH (version 11.0.667). α-Diversity indices were calculated with Mothur (version 3.8.31), β-diversity was analyzed through ANOVA and principal coordinate analysis (PCoA), and microbial community functions were predicted using PICRUSt2 (version 2.5.2).

### 2.7. Determination of Hyaluronidase Inhibition Rate

The bacterial suspension was prepared by culturing the strain to stationary phase in MRS (Qingdao Hopebio Biotechnology Co., Ltd., Qingdao, China) broth supplemented with L-cysteine (Macklin Biochemical Technology Co., Ltd., Shanghai, China) [26]. To each tube, 0.1 mL of 2.5 mmol/L CaCl_2_ (Macklin Biochemical Technology Co., Ltd., Shanghai, China) and 0.5 mL of 600 U/mL hyaluronidase (Macklin Biochemical Technology Co., Ltd., Shanghai, China) were added, followed by incubation at 37 °C for 20 min. Subsequently, 0.5 mL of the bacterial suspension was added and the tubes were incubated at 37 °C for another 20 min. Then, 0.5 mL of 0.5 mg/mL sodium hyaluronate solution was added, and the mixture was further incubated at 37 °C for 30 min. After standing at room temperature for 5 min, 0.5 mL of acetylacetone solution (freshly prepared by dissolving 1.4 mL acetylacetone (Aladdin Biochemical Technology Co., Ltd., Shanghai, China) in 20 mL of 1.0 mol/L Na_2_CO_3_ (Sinopharm Chemical Reagent Co., Ltd., Shanghai, China) solution) and 0.1 mL of 0.4 mol/L NaOH (Macklin Biochemical Technology Co., Ltd., Shanghai, China) were added. The tubes were then placed in a boiling water bath for 15 min, immediately transferred to an ice bath for 5 min, followed by addition of 1 mL of Ehrlich’s reagent, prepared by dissolving 0.8 g *p*-dimethylaminobenzaldehyde (Macklin Biochemical Technology Co., Ltd., Shanghai, China) in 15 mL concentrated hydrochloric acid and 15 mL absolute ethanol (Macklin Biochemical Technology Co., Ltd., Shanghai, China). After color development at room temperature for 20 min, absorbance was measured at 530 nm.

### 2.8. Cell Culture and Determination of β-Hexosaminidase Inhibition Rate

Bacterial cells were cultured to the logarithmic growth phase. The culture was then centrifuged at 4 °C and 6000× *g* for 8 min to collect the supernatant. The supernatant was filter-sterilized, adjusted to pH 7.0 using sterile NaOH solution, and prepared as a sterile fermentation supernatant for subsequent use [27]. The rat basophilic leukemia cell line RBL-2H3 (Cell Bank of the Chinese Academy of Sciences, Shanghai, China) was thawed and cultured in complete DMEM (Gibco, Thermo Fisher Scientific, Paisley, UK) supplemented with 15% fetal bovine serum(Gibco, Thermo Fisher Scientific, Paisley, UK) and 1% penicillin-streptomycin (Macklin Biochemical Technology Co., Ltd., Shanghai, China) at 37 °C under 5% CO_2_, with the medium replaced every two days. Upon reaching 80–90% confluency, cells were passaged by washing twice with sterile PBS (Biosharp, Beijing, China), digesting with 0.25% trypsin-EDTA (Gibco, Grand Island, NY, USA), and neutralizing the digestion with DMEM containing 10% fetal bovine serum. The cell suspension was centrifuged at 1000× *g* for 5 min, resuspended in fresh complete DMEM, and seeded into new culture flasks. After three consecutive passages, cells exhibiting stable growth were used for subsequent experiments.

RBL-2H3 cells were seeded into 96-well plates at a density of 2 × 10^5^ cells/mL. Cells were sensitized by adding 200 μL of 0.5 μg/mL anti-DNP-IgE (Sigma-Aldrich, St. Louis, MO, USA) antibody per well and incubating overnight at 4 °C. After removing the sensitization solution and washing three times with PBS, 200 μL of the prepared sterile fermentation supernatant was added to each well and incubated at 37 °C for 1 h. Following removal of the supernatant and three PBS washes, 100 μL of 1 μg/mL DNP-HSA (Sigma-Aldrich, St. Louis, MO, USA) antigen was added to each well and incubated at 37 °C for 20 min to stimulate degranulation. The supernatant from each well was collected, and the inhibitory effect of the fermentation supernatant on β-hexosaminidase release was measured using a rat β-hexosaminidase ELISA kit (Nanjing Jiancheng Bioengineering Institute, Nanjing, China) according to the manufacturer’s instructions.

### 2.9. Statistical Analysis

All statistical analyses and graph generation were performed using GraphPad Prism 9.0 software. Normality of residuals was assessed using the Shapiro–Wilk test, and homogeneity of variances was assessed using Levene’s test. For data meeting the assumptions of parametric analysis, differences between groups were assessed by analysis of variance (one-way or two-way ANOVA, as appropriate), followed by Tukey’s HSD test for multiple comparisons. For data that did not meet these assumptions, nonparametric tests (Kruskal–Wallis test followed by Dunn’s post hoc test) were applied. Statistical significance is denoted as follows: * *p* < 0.05, ** *p* < 0.01, *** *p* < 0.001, **** *p* < 0.0001; NS indicates no significant difference.

## 3. Results

### 3.1. Strain-Specific Amelioration of AD-like Symptoms in Mice by L. rhamnosus

**Figure 2:**
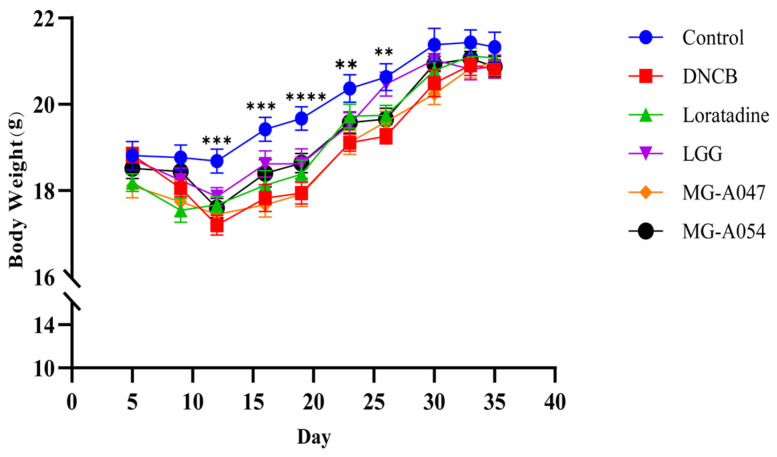
Effects of Different Treatment Groups on Body Weight in Mice with AD. Data are presented as mean ± SEM. ** *p* < 0.01, *** *p* < 0.001, **** *p* < 0.0001 vs. DNCB group.

Body weight was monitored throughout the study. From days 12 to 26, mice in the model group showed significantly lower body weight than those in the control group. Although all treatment groups exhibited a non-significant increasing trend in body weight compared to the model group, the results indicate that none of the interventions—loratadine, *L. rhamnosus* GG, MG-A047, or MG-A054—adversely affected normal growth (Figure 2).

Clinical assessment of AD symptoms indicated that topical application of 1% DNCB (days 8–14) induced pronounced erythema on the dorsal skin and right ear of mice in the model group. By the mid-experimental phase, the dorsal lesions had progressed to diffuse erythematous patches with notable crust formation. Concurrently, mice in the model group exhibited frequent and persistent scratching behavior directed toward the affected areas. In contrast, all treatment groups demonstrated measurable reductions in both clinical lesion severity and scratching activity.

**Figure 3:**
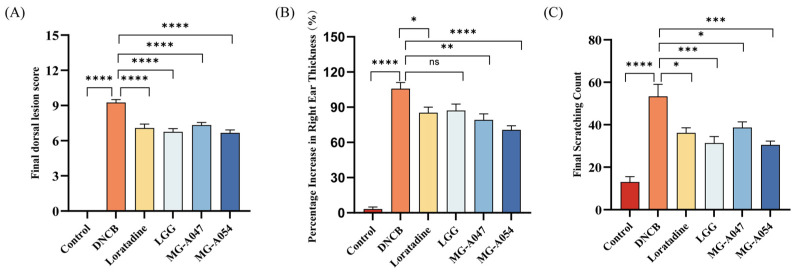
Effect of *L. rhamnosus* on symptom severity in DNCB-induced AD mice: (**A**) Dorsal lesion score (*n* = 12). (**B**) Percent increase in right ear thickness (*n* = 12). (**C**) Final scratching frequency (*n* = 6). Data are presented as mean ± SEM. * *p* < 0.05, ** *p* < 0.01, *** *p* < 0.001, **** *p* < 0.0001 vs. DNCB group.

At the experimental endpoint (day 35), skin lesion severity was evaluated. DNCB-treated mice showed significantly increased dorsal lesion severity compared to the control group. All treatment groups significantly reduced lesion severity relative to the DNCB group (*p* < 0.0001; Figure 3A). The mean improvement in lesion severity in the MG-A054 group was superior to that in both the loratadine and LGG groups, although the intergroup differences did not reach statistical significance (*p* > 0.05). Representative photographs of dorsal skin lesions are shown in Supplementary Figure S1. In parallel, ear edema was assessed by measuring the increase in right ear thickness from baseline at the endpoint. The model group exhibited a significantly greater increase in right ear thickness compared to the control group. Compared with the model group, treatments with loratadine, MG-A047, and MG-A054 (but not LGG) significantly attenuated the increase in ear thickness and effectively alleviated DNCB-induced ear swelling (*p* < 0.05; Figure 3B). Moreover, the increases in ear thickness observed in the MG-A047 and MG-A054 groups were lower than those in both the LGG and loratadine groups. Consistent with these macroscopic improvements, a reduction in disease-associated scratching behavior was also observed. Compared to the control group, DNCB-treated mice displayed a significant increase in scratching frequency. All treatment groups significantly reduced the scratching count and alleviated pruritus severity (*p* < 0.05; Figure 3C). Among them, the MG-A054 group showed a greater mean improvement in the scratching index compared to both the LGG and loratadine groups, although the intergroup differences were not statistically significant.

### 3.2. Strain-Specific Amelioration of Histopathological Phenotypes in Mice with AD by L. rhamnosus

**Figure 4:**
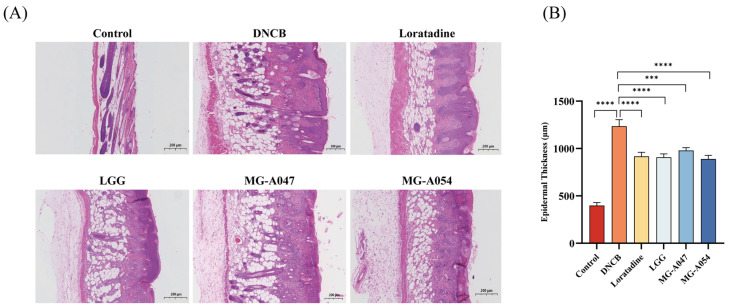
Effect of *L. rhamnosus* on skin pathology in DNCB-induced AD mice: (**A**) Representative H&E-stained sections of dorsal skin (scale bar = 200 μm). (**B**) Epidermal thickness quantification (*n* = 6 per group). Data are presented as mean ± SEM. *** *p* < 0.001, **** *p* < 0.0001 vs. DNCB group.

Histopathological evaluation was performed on dorsal skin tissues using hematoxylin and eosin (H&E) staining. Compared with the control group, the DNCB-treated model group exhibited parakeratosis, crust formation, significant epidermal hyperplasia, and marked inflammatory cell infiltration in the dermis. Relative to the DNCB model group, all *L. rhamnosus* treatment groups and the loratadine group showed significant reductions in both epidermal thickness and inflammatory cell infiltration (*p* < 0.0001; Figure 4A,B). Histopathological scoring indicated that the mean improvement in the MG-A054 group was slightly greater than that in both the LGG and loratadine groups, although the intergroup differences did not reach statistical significance.

### 3.3. Strain-Specific Modulation of Mast Cell and Eosinophil Infiltration in AD Mice by L. rhamnosus

**Figure 5:**
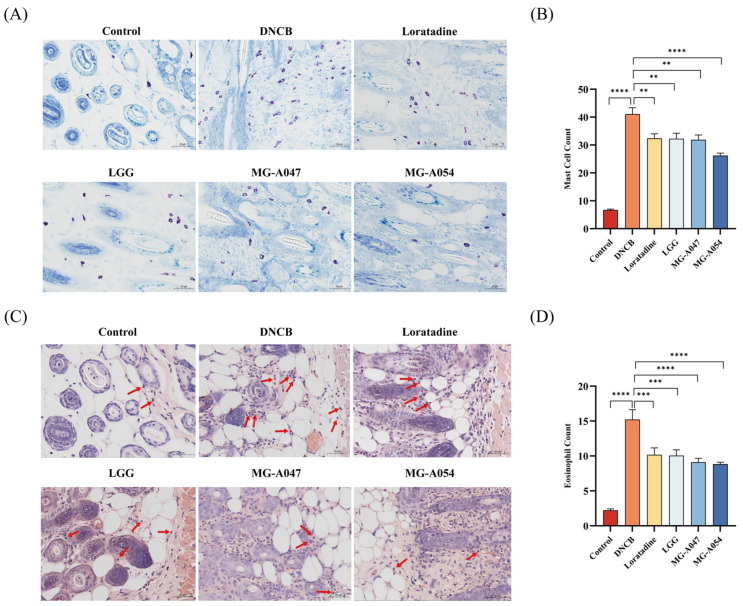
Effect of *L. rhamnosus* on skin mast cells and eosinophils in DNCB-induced AD mice. (**A**) Representative toluidine blue-stained sections. (**B**) Mast cell count (*n* = 6 per group). (**C**) Representative Congo red-stained sections. (**D**) Eosinophil count (*n* = 6 per group). Data are presented as mean ± SEM. ** *p* < 0.01, *** *p* < 0.001, **** *p* < 0.0001 vs. DNCB group. The arrow points to stained eosinophils.

Toluidine blue staining showed a significant increase in mast cell numbers in the skin of DNCB-treated mice compared to the control group (*p* < 0.0001; Figure 5A,B). Both probiotic treatments and loratadine significantly suppressed this increase (*p* < 0.05; Figure 5A,B), with the two *L. rhamnosus* strains showing stronger inhibitory effects than LGG and loratadine. Congo red staining further indicated that DNCB induction resulted in a marked elevation in eosinophil counts. All intervention groups significantly reduced eosinophil infiltration (*p* < 0.05; Figure 5C,D). Eosinophil counts in the MG-A047 and MG-A054 groups were observed to be lower than those in the LGG and loratadine groups, but the differences did not reach statistical significance.

### 3.4. Strain-Specific Modulation of Serum IgE and IL-4 Levels in AD Mice by L. rhamnosus

**Figure 6:**
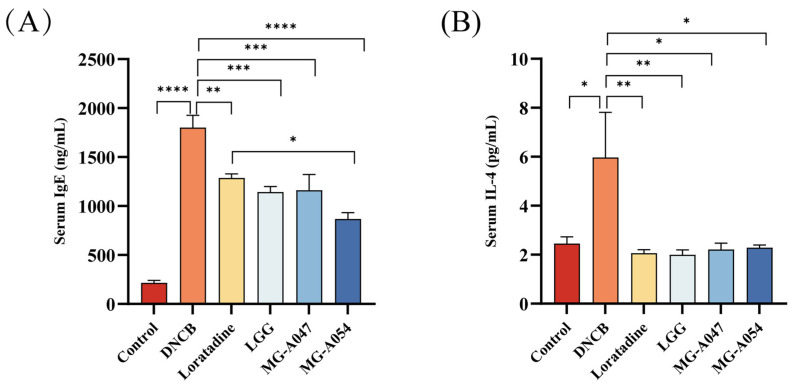
Effect of *L. rhamnosus* on serum IgE and IL-4 in DNCB-induced AD mice: (**A**) Total serum IgE concentration. (**B**) Serum IL-4 concentration. Data are presented as mean ± SEM. * *p* < 0.05, ** *p* < 0.01, *** *p* < 0.001, **** *p* < 0.0001 vs. DNCB group.

DNCB treatment significantly increased serum total IgE and IL-4 levels in mice relative to the control group. Following intervention with *L. rhamnosus* strains, the loratadine, LGG, MG-A054, and MG-A047 groups all exhibited significantly reduced serum IgE and IL-4 levels compared to the DNCB model group (*p* < 0.05; Figure 6A,B). Furthermore, the reduction in serum IgE in the MG-A054 group was significantly greater than that in the loratadine group (*p* < 0.05).

### 3.5. Effects of L. rhamnosus Supplement on the Gut Microbiota in Mice with AD

**Figure 7:**
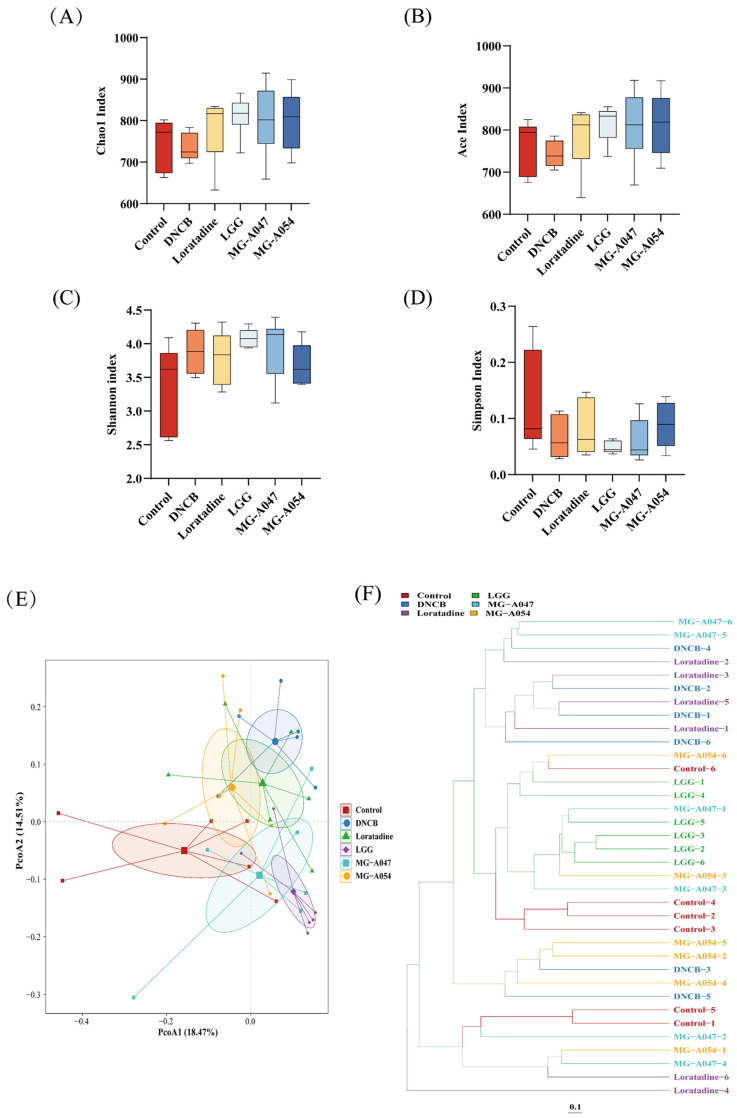
Effect of *L. rhamnosus* on gut microbiota in DNCB-induced AD mice: (**A**) Chao1 index, (**B**) Ace index, (**C**) Shannon index, (**D**) Simpson index, (**E**) β-diversity PCoA analysis. The vertical and horizontal dashed lines represent the zero lines of PCoA1 and PCoA2, respectively, (**F**) β-diversity cluster tree analysis. The scale bar of 0.1 indicates the level of dissimilarity between bacterial communities. Data are expressed as the mean ± SEM (*n* = 6 per group).

The fecal microbiota was assessed via 16S rRNA gene amplicon sequencing to evaluate the effects of loratadine and three *L. rhamnosus* strains. Gut microbiota α-diversity was measured using the Shannon index, Chao1 estimator, and Ace estimator (Figure 7A–D). All three *L. rhamnosus* intervention groups showed higher Shannon indices compared to the control, indicating that probiotic treatment enhanced gut microbial diversity. Similar increases were observed in the Chao1 and Ace richness estimates. In β-diversity analysis, Principal Coordinates Analysis (PCoA) and hierarchical clustering dendrograms revealed clear separation between the control and DNCB model groups, with non-overlapping confidence ellipses. In contrast, the loratadine group and the three probiotic groups showed distinct microbial community structures, suggesting different mechanisms underlying microbiota modulation among these interventions. After intervention with *L. rhamnosus* strains MG-A054, MG-A047, and LGG, the microbial communities of these groups clustered closer to the control in multivariate space (Figure 7E,F). PERMANOVA (Adonis) confirmed that DNCB induction significantly altered gut microbiota structure relative to the control (R^2^ = 0.27, *p* = 0.05). MG-A054 intervention partially reversed this dysbiosis, shown by a significant difference in community structure compared to the DNCB model group (R^2^ = 0.19, *p* = 0.10). Furthermore, the microbiota of the MG-A054 group resembled that of the control group more closely (R^2^ = 0.08, *p* = 0.45).

**Figure 8:**
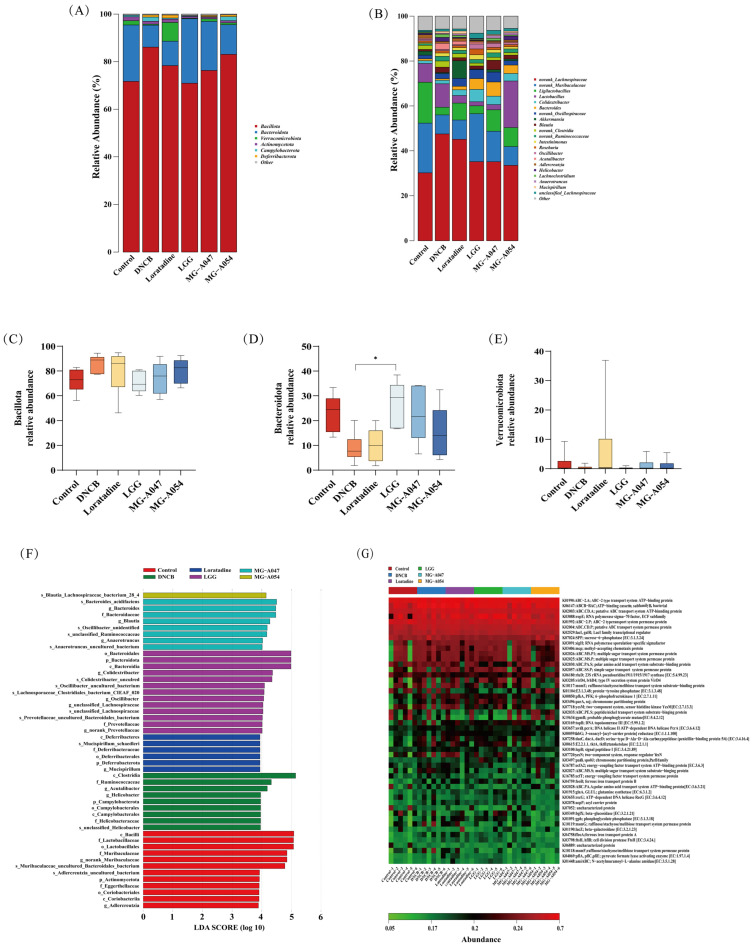
Effect of *L. rhamnosus* on gut microbiota in DNCB-induced AD mice: (**A**) Phylum-level composition, (**B**) Genus-level composition, (**C**) Relative abundance of Bacillota, (**D**) Relative abundance of Bacteroidota, (**E**) Relative abundance of Verrucomicrobiota, (**F**) LEfSe analysis identifying differential taxa, (**G**) Heatmap of predicted KEGG pathways. Data are expressed as the mean ± SEM (*n* = 6 per group). * *p* < 0.05, vs. DNCB group.

At the phylum level, compared with the control, the DNCB model group showed significantly higher relative abundances of Campylobacterota and Bacillota, but lower abundances of Bacteroidota and Verrucomicrobiota. Intervention with loratadine, *L. rhamnosus* MG-A047, or MG-A054 partially restored the relative abundance of Verrucomicrobiota. At the genus level, the abundance of *Bacteroides* was elevated in the loratadine group and all three *L. rhamnosus* intervention groups relative to the DNCB group. The MG-A054 group exhibited substantially higher abundances of *Lactobacillus* and *Ligilactobacillus*, whereas *Lactobacillus* levels were lower in the MG-A047 and LGG groups. The abundance of *Lachnospira* in the MG-A054 group approached that of the control group. Notably, *Akkermansia* abundance was higher in the loratadine, MG-A047, and MG-A054 groups than in the DNCB model group, with the greatest increase observed in the loratadine group (Figure 8A–E). LEfSe analysis identified 28 differentially abundant microbial taxa among the four intervention groups. The loratadine group was enriched in *Mucispirillum* (Deferribacterota); the LGG group was dominated by Bacteroidota, including Prevotellaceae and *Colidextribacter*; and the MG-A047 group showed the highest abundances of *Blautia* and *Bacteroides*. Functional prediction of cecal contents from AD mice was conducted using PICRUSt2 with the KEGG database. The results indicated that each intervention group differentially modulated the DNCB-induced functional dysbiosis of the gut microbiota. The predicted functional profile of the MG-A054 group most closely resembled that of the control, suggesting that this strain exerted the strongest restorative effect on predicted gut microbial metabolic functions. Specifically, the MG-A054 group exhibited enriched functional genes associated with protein-tyrosine-phosphatase, sucrose-6-phosphatase, peptide/nickel transport system, 23S rRNA pseudouridine synthase, and phosphoglycerate mutase. In contrast, the LGG and MG-A047 groups showed higher predicted abundances of genes encoding RNA polymerase sigma-70 factor and beta-glucosidase (Figure 8F,G).

### 3.6. In Vitro Inhibition of Hyaluronidase and β-Hexosaminidase Activity in RBL-2H3 Cells by L. rhamnosus

**Figure 9:**
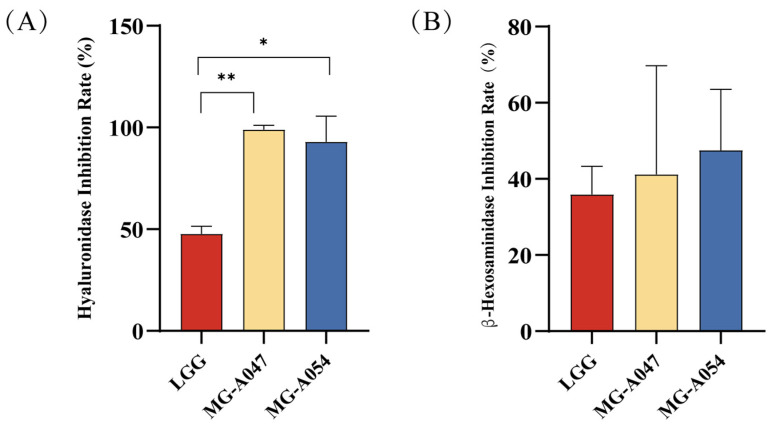
In vitro inhibition of key AD-related targets by *L. rhamnosus* strains: (**A**) Inhibition of hyaluronidase activity by bacterial cell suspensions. (**B**) Suppression of β-hexosaminidase release from RBL-2H3 mast cells by bacterial culture supernatants. Data are presented as mean ± SEM. * *p* < 0.05, ** *p* < 0.01 vs. DNCB group.

In AD, hyaluronidase drives inflammation and pruritus through hyaluronic acid degradation, resulting in skin barrier disruption and the production of pro-inflammatory fragments. Assessing the hyaluronidase-inhibitory activity of probiotics provides direct evidence for their potential therapeutic mechanism: preserving skin barrier integrity and interrupting the inflammatory cascade. The hyaluronidase inhibition rates of three selected *L. rhamnosus* strains were evaluated. The results showed that both *L. rhamnosus* MG-A047 and MG-A054 had significantly stronger hyaluronidase inhibitory activity than LGG (*p* < 0.05; Figure 9A). In addition to hyaluronidase, β-hexosaminidase (β-Hex) released from degranulating mast cells and basophils is another key mediator of pruritus and inflammation in AD. To examine whether the culture supernatants of the three *L. rhamnosus* strains could affect β-Hex release, RBL-2H3 cells were sensitized with anti-DNP-IgE and stimulated with DNP-BSA. The results showed that although there were numerical differences in the inhibition rates of β-hexosaminidase release among the supernatants of the three *L. rhamnosus* strains, none reached statistical significance (Figure 9B).

## 4. Discussion

AD is a chronic inflammatory skin disease characterized by impaired skin barrier function and aberrant immune responses [28]. Current therapeutic approaches, such as topical corticosteroids, calcineurin inhibitors and biologics, primarily target the suppression of overactive immune responses to alleviate symptoms [29,30]. However, long-term use of these treatments can be associated with adverse effects and offers limited efficacy in addressing the underlying etiology of the disease. Emerging evidence suggests that probiotics may represent a promising novel strategy for AD management by modulating the gut–skin axis, improving immune regulation, and promoting skin barrier repair [31]. Evaluation of three *L. rhamnosus* strains in a DNCB-induced AD model revealed that MG-A054 conferred significant therapeutic benefits, including reduced skin inflammation, ear edema, and scratching behavior. Its efficacy outperformed that of loratadine, thereby affirming its therapeutic advantage and the functional importance of strain-specificity among probiotics. Histopathological examination further demonstrated the tissue-protective effects of MG-A054, evidenced by markedly reduced epidermal hyperplasia and dermal infiltration of inflammatory cells (notably mast cells and eosinophils). These cells are central to AD pathogenesis, whose activation and degranulation directly contribute to pruritus, vasodilation, and tissue damage. The decreased cellular infiltration observed here implies that the probiotic may locally alleviate inflammation and itching by stabilizing these cells and inhibiting their activation and recruitment. This aligns with existing literature on the protective mechanisms of probiotics in AD [32]. Moreover, the differential effects among the strains indicate that distinct *L. rhamnosus* strains may employ unique mechanisms to stabilize effector cells and achieve local anti-inflammatory and antipruritic outcomes.

As a hapten, 2,4-dinitrochlorobenzene (DNCB) induces an immune-pathological process analogous to human AD by directly compromising the skin barrier and eliciting a robust delayed-type hypersensitivity response. The DNCB-induced murine model of AD effectively recapitulates core pathological features of AD, such as impaired skin barrier function, excessive activation of Th2-mediated immunity, and the manifestation of pruritic behaviors, thereby serving as a validated experimental platform for AD investigation [33,34]. Serum immunoglobulin E (IgE) is a pivotal biomarker in AD, essential for both diagnosis and monitoring of disease progression. Clinical studies have established a positive correlation between elevated total serum IgE levels and AD severity, driven in part by Th2-associated cytokines, including IL-4 and IL-13, which directly stimulate IgE synthesis in B lymphocytes [35,36]. Consistent with this, mice in the DNCB-induced model group displayed markedly increased IgE levels, indicative of the characteristic Th2-polarized immune profile observed in AD. Administration of distinct *L. rhamnosus* strains via oral gavage significantly lowered serum total IgE concentrations. Notably, *L. rhamnosus* MG-A054 exerted the most pronounced suppressive effect, surpassing that of the positive control agent loratadine-a finding of particular interest. IL-4 plays a central role in AD pathogenesis by orchestrating the Th2-type immune response, promoting IgE class-switching in B cells, and disrupting epidermal barrier integrity. In AD, IL-4 directly suppresses the expression of key barrier proteins like filaggrin, enhances transepidermal water loss, and activates eosinophils and mast cells, thereby aggravating pruritus and cutaneous inflammation. Consequently, IL-4 represents a critical therapeutic target in AD management [37]. Serum IL-4 levels were quantified using an IL-4-specific cytometric bead array (CBA). The analysis revealed that treatment with *L. rhamnosus* MG-A047, MG-A054, or LGG led to a reduction in this pro-inflammatory cytokine, albeit with varying potency across strains. These observations align with existing literature on probiotic-mediated immunomodulation in AD [38,39].

This study confirmed that specific strains of *L. rhamnosus*, especially MG-A054, achieved multi-target amelioration of AD pathology by modulating Th2-type immune polarization, reducing key effector molecules like IgE and IL-4, and directly inhibiting mast cell degranulation. Its therapeutic efficacy was superior to that of the comparator strains LGG and MG-A047, which is consistent with prior reports on the high strain-specificity of probiotic functions [19,40]. This specificity was manifested not only in the extent of symptom alleviation but also in differential modulation of the host gut microecology. Gut microbiota data provided key supportive evidence: DNCB induction disrupted both the skin barrier and immune balance, increased the abundance of potentially harmful Campylobacterota, decreased beneficial Verrucomicrobiota, and resulted in gut dysbiosis. Although all probiotic interventions partially alleviated the dysbiosis, their modulation patterns were distinct. At the genus level, only MG-A054 significantly and specifically increased Lactobacillus abundance, whereas MG-A047 and LGG lacked this effect, suggesting that unique colonization or growth-promoting capacity may explain its superior efficacy. LEfSe analysis further identified strain-specific biomarkers: the MG-A054 and MG-A047 groups were characterized by enrichment of short-chain fatty acid (SCFA)-producing genera such as *Blautia*, whereas the LGG group showed enrichment of *Colidextribacter*. Given that SCFAs have been demonstrated to suppress Th2-type immune responses and reduce IgE production via activation of GPR41/GPR43 receptors on immune cells, the enrichment of SCFA-producing genera in the MG-A054 and MG-A047 groups may represent a key mechanism underlying their therapeutic efficacy in ameliorating AD. This also indicates that different strains may modulate the host by establishing distinct ecological niches. PICRUSt2-based KEGG functional prediction offered further evidence for strain-specificity at the functional potential level. Predictions indicated that MG-A054 specifically enriched metabolic pathways involved in epidermal repair, including protein-tyrosine phosphatase activity. In contrast, LGG and MG-A047 displayed distinct functional enrichment patterns, such as pathways related to polysaccharide metabolism. Notably, only the MG-A054 group exhibited a predicted functional profile that closely resembled that of the healthy controls, indicating that this strain mediated functional recalibration of the dysbiotic microbiota, extending beyond structural reshaping. This unique functional reprogramming capacity may modulate the host immune microenvironment via strain-specific microbial metabolite profiles.

The findings of this study suggest that the marked efficacy of MG-A054 may be attributed to its systemic modulatory role within the gut–skin axis. This strain exhibits a degree of selectivity in promoting the proliferation of *Lactobacillus* and enriching SCFAs-producing bacterial populations, accompanied by a trend toward structural and functional reprogramming of the gut microbiota toward a healthier state, which may contribute to systemic immune modulation (e.g., reduction in serum IgE and IL-4). This process aligns with findings from studies showing that gut-derived metabolites can modulate distal inflammatory responses [20,21,41]. In vitro assays further demonstrated that MG-A054 directly inhibits hyaluronidase activity and mast cell degranulation, consistent with the observed improvements in skin barrier integrity and alleviation of pruritus in vivo. The combined intestinal and cutaneous effects contribute to the multi-target therapeutic profile of MG-A054, which may explain its superior efficacy compared with LGG and MG-A047. Notably, although LGG exhibited lower in vitro inhibitory activity against hyaluronidase than MG-A047 and MG-A054, it showed better overall efficacy in the animal model than MG-A047. This observation underscores the potentially distinct modes of action among different strains of *L. rhamnosus*: LGG may compensate for its relatively weaker local bioactivity by enhancing gut microbial diversity or activating alternative immune pathways. Therefore, the evaluation of probiotics for AD should concurrently consider both local bioactivity and systemic regulatory potential. A multidimensional assessment framework will enable a more comprehensive exploration of their underlying mechanisms and provide a scientific basis for clinical translation. Moreover, these results further highlight the importance of implementing strain-level precision screening in the development of probiotic-based therapies [42,43,44].

There are several aspects of this study that could be optimized in future research. First, the sample size was relatively small, which precluded quantitative analysis of key metabolites such as short-chain fatty acids, thereby limiting in-depth interpretation of the gut–skin axis at the metabolic level; in addition, dynamic changes in the local skin microbiota were not monitored. Second, the findings presented here are preclinical and were obtained using a DNCB-induced murine model of atopic dermatitis. Although this model offers experimental utility, it has certain limitations in fully recapitulating the pathogenesis of human disease, as its acute elicitation nature does not completely reflect the chronic, recurrent immune microenvironment characteristic of human AD. Furthermore, in future studies, it will be important to extend the analysis beyond the IL-4 pathway to include other key mediators involved in atopic dermatitis pathogenesis. In particular, incorporating pruritus-related mediators such as IL-31, along with evaluating regulatory immune cell populations, could yield further valuable insights. Subsequent investigations may employ models with stronger Th2 polarization (e.g., MC903) and integrate approaches such as metabolomic profiling, fecal microbiota transplantation, and multi-model comparisons to systematically elucidate the mechanisms of action of probiotic interventions and facilitate their clinical translation.

## 5. Conclusions

By systematically comparing the therapeutic effects of three *L. rhamnosus* strains in a DNCB-induced mouse model of AD, this study reveals pronounced strain-specificity in probiotic-mediated alleviation of AD. The MG-A054 strain exhibited the most comprehensive overall efficacy, which likely stems from its multifaceted regulatory actions, including correcting the Th2-skewed immune response by lowering serum total IgE and IL-4 levels, preserving skin barrier function through inhibition of hyaluronidase activity, alleviating pruritus by stabilizing mast cells and reducing the release of itch-inducing mediators, and beneficially reshaping the gut microbiota to maintain immune homeostasis. Collectively, these findings suggest that MG-A054 likely ameliorates AD by coordinating the gut–skin axis and related pathways, positioning it as a candidate strain for targeted improvement of AD. However, the active metabolites and causal pathways through which MG-A054 exerts its effects remain unclear. Subsequent studies could integrate approaches such as fecal microbiota transplantation and metabolomic profiling to further investigate and validate these mechanisms, thereby laying a mechanistic foundation for future clinical translation.

## Data Availability

The original contributions presented in this study are included in the article. Further inquiries can be directed to the corresponding authors.

## Supplementary Materials

The following supporting information can be downloaded at: https://www.mdpi.com/article/10.3390/nu18091335/s1, Figure S1. Representative photographs of dorsal skin lesions in each group. Table S1. Exact *p*-values for data presented in Figure 3. Table S2. Exact *p*-values for data presented in Figure 4. Table S3. Exact *p*-values for data presented in Figure 5. Table S4. Exact *p*-values for data presented in Figure 6. Table S5. Exact *p*-values for data presented in Figure 8 and Figure 9.

## Abbreviations

The following abbreviations are used in this manuscript:

AD	Atopic Dermatitis
DNCB	2,4-dinitrochlorobenzene
IL-4	Interleukin-4
IL-13	Interleukin-13
IgE	Immunoglobulin E
IL-10	Interleukin-10
IL-17	Interleukin-17
IL-31	Interleukin-31
IFN-γ	Interferon-γ
TGF-β	Transforming Growth Factor-β
MRS	Man Rogosa Sharpe Medium
β-Hex	β-Hexosaminidase
SCFAs	Short-Chain Fatty Acids
