# Supplementary material for: Therapeutic Potential of Specific Lacticaseibacillus rhamnosus Strains for DNCB-Induced Atopic Dermatitis in Mice
Source: Nutrients. 2026 Apr 23;18(9):1335. doi: 10.3390/nu18091335 (PMC13164683; doi:10.3390/nu18091335)

Supplementary Table S1. Exact p-values for data presented in Figure 3.

Figure panel	Comparison	p-value
3A	Control vs. DNCB	<0.0001
3A	DNCB vs. Loratadine	<0.0001
3A	DNCB vs. LGG	<0.0001
3A	DNCB vs. MG-A047	<0.0001
3A	DNCB vs. MG-A054	<0.0001
3B	Control vs. DNCB	<0.0001
3B	DNCB vs. Loratadine	0.0242
3B	DNCB vs. LGG	0.0538
3B	DNCB vs. MG-A047	0.0013
3B	DNCB vs. MG-A054	<0.0001
3C	Control vs. DNCB	<0.0001
3C	DNCB vs. Loratadine	0.0102
3C	DNCB vs. LGG	0.0006
3C	DNCB vs. MG-A047	0.0380
3C	DNCB vs. MG-A054	0.0004

Supplementary Table S2. Exact p-values for data presented in Figure 4.

Figure panel	Comparison	p-value
4B	Control vs. DNCB	<0.0001
4B	DNCB vs. Loratadine	<0.0001
4B	DNCB vs. LGG	<0.0001
4B	DNCB vs. MG-A047	0.0007
4B	DNCB vs. MG-A054	<0.0001

Supplementary Table S3. Exact p-values for data presented in Figure 5.

Figure panel	Comparison	p-value
5B	Control vs. DNCB	<0.0001
5B	DNCB vs. Loratadine	0.0027
5B	DNCB vs. LGG	0.0023
5B	DNCB vs. MG-A047	0.0012
5B	DNCB vs. MG-A054	<0.0001
5D	Control vs. DNCB	<0.0001
5D	DNCB vs. Loratadine	0.0005
5D	DNCB vs. LGG	0.0003
5D	DNCB vs. MG-A047	<0.0001
5D	DNCB vs. MG-A054	<0.0001

Supplementary Table S4. Exact p-values for data presented in Figure 6.

Figure panel	Comparison	p-value
6A	Control vs. DNCB	<0.0001
6A	DNCB vs. Loratadine	0.0035
6A	DNCB vs. LGG	0.0001
6A	DNCB vs. MG-A047	0.0002
6A	DNCB vs. MG-A054	<0.0001
6A	Loratadine vs. MG-A054	0.0289
6B	Control vs. DNCB	0.0246
6B	DNCB vs. Loratadine	0.0086

6B	DNCB vs. LGG	0.0072
6B	DNCB vs. MG-A047	0.0130
6B	DNCB vs. MG-A054	0.0158

Supplementary Table S5. Exact p-values for data presented in Figure 8 and Figure 9.

Figure panel	Comparison	p-value
8D	DNCB vs. LGG	0.0128
9A	LGG vs. MG-A047	0.0081
9A	LGG vs. MG-A054	0.0146

Supplementary Figure S1. Representative photographs of dorsal skin lesions in each group.

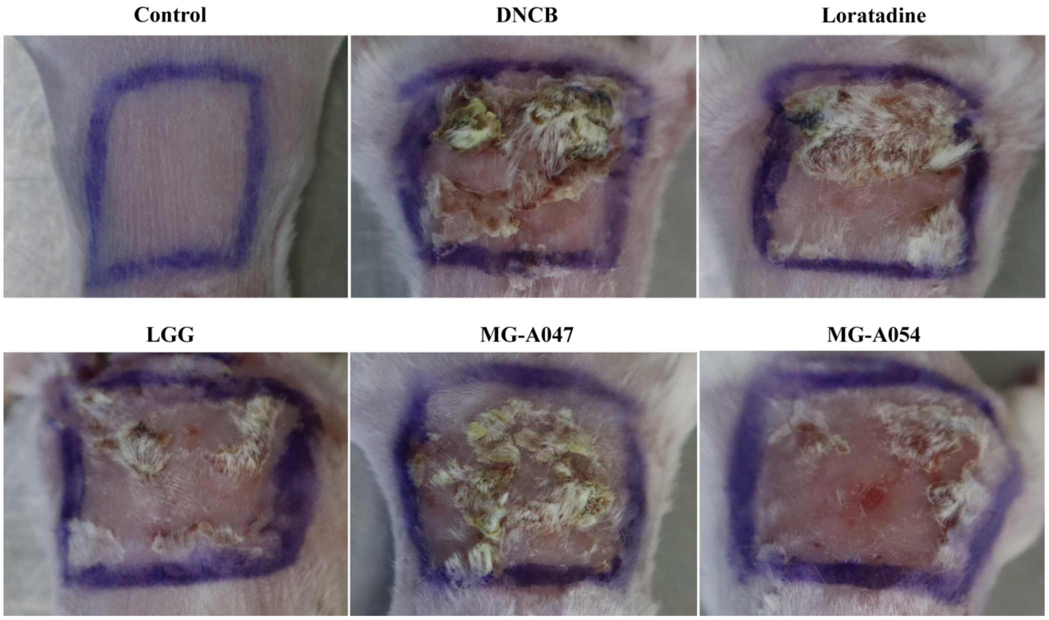

Supplement: Supplementary file 1 [file nutrients-18-01335-s001.zip › nutrients-4208382-supplementary.pdf]
